# Sharing electronic and ionic transfer channels for high-energy-density and stable quasi-solid-state lithium-oxygen battery

**DOI:** 10.1093/nsr/nwag134

**Published:** 2026-03-05

**Authors:** Yuanguo Wu, Zhuojun Zhang, Jiaqi Wang, Hongtao Qu, Jing Li, Liuxi Yang, Amanda Kale, Xikun Zhang, Xiangyu Wen, Zhihong Wang, Zhe Lü, Yu Li, Peng Tan, Xingbao Zhu, Bao-Lian Su

**Affiliations:** Laboratory of Inorganic Materials Chemistry (CMI), University of Namur, Namur B-5000, Belgium; School of Physics, Harbin Institute of Technology, Harbin 150000, China; Gotion High-tech Co., Ltd., Hefei 230026, China; Department of Thermal Science and Energy Engineering, University of Science and Technology of China, Hefei 230000, China; School of Physics, Harbin Institute of Technology, Harbin 150000, China; Laboratory of Inorganic Materials Chemistry (CMI), University of Namur, Namur B-5000, Belgium; Laboratory of Inorganic Materials Chemistry (CMI), University of Namur, Namur B-5000, Belgium; Laboratory of Inorganic Materials Chemistry (CMI), University of Namur, Namur B-5000, Belgium; Laboratory of Inorganic Materials Chemistry (CMI), University of Namur, Namur B-5000, Belgium; Laboratory of Inorganic Materials Chemistry (CMI), University of Namur, Namur B-5000, Belgium; School of Physics, Harbin Institute of Technology, Harbin 150000, China; School of Physics, Harbin Institute of Technology, Harbin 150000, China; School of Physics, Harbin Institute of Technology, Harbin 150000, China; State Key Laboratory of Advanced Technology for Materials Synthesis and Processing, Wuhan University of Technology, Wuhan 430070, China; Department of Thermal Science and Energy Engineering, University of Science and Technology of China, Hefei 230000, China; School of Physics, Beijing Institute of Technology, Beijing 102488, China; Laboratory of Inorganic Materials Chemistry (CMI), University of Namur, Namur B-5000, Belgium; State Key Laboratory of Advanced Technology for Materials Synthesis and Processing, Wuhan University of Technology, Wuhan 430070, China

**Keywords:** quasi-solid-state lithium-air battery, gel polymer electrolyte, graphene aerogel, electrochemistry-loading transport coupling

## Abstract

Thick cathodes are essential for practical high-energy batteries, yet their development is hindered by sluggish charge kinetics, particularly in lithium-oxygen batteries (LOBs) where robust three-phase boundaries (TPBs) for e^−^, Li^+^, and O_2_ are indispensable. Herein, we propose a gel polymer electrolyte (GPE) integration strategy that enables the construction of a streamlined dual-conductive network for both e^−^ and Li^+^ while preserving optimal porosity for rapid O_2_ diffusion in thick cathodes (∼2 mm). This innovative architecture creates extensive and continuous TPBs throughout the entire cathode, enabling an exceptional areal capacity of 34.6 mAh cm^−2^, surpassing most previously reported LOBs, and a record-breaking gravimetric capacity of 19 000 mAh g^−1^. Numerical simulations further validate the superiority of this approach. Our work provides a proof of concept for overcoming kinetic transport limitations in thick cathodes, paving the way for next-generation high-capacity and stable LOBs.

## Teaser

A tailor-made, ion-conductive sheath created via in-situ gel electrolyte solidification simultaneously reinforces the porous thick cathode and unlocks its high capacity–charting a pathway toward high-energy lithium-oxygen batteries.

## INTRODUCTION

Demands for an improved range of electric vehicles necessitate the development of more robust batteries with higher energy densities, surpassing the theoretical limitation of commercially used lithium-ion batteries [1,2]. Lithium-oxygen batteries (LOBs), with an exceptional theoretical energy density of 3500 Wh kg^−1^, have consequently garnered significant attention and are considered as the most promising candidates [3,4].

**Figure 1.**
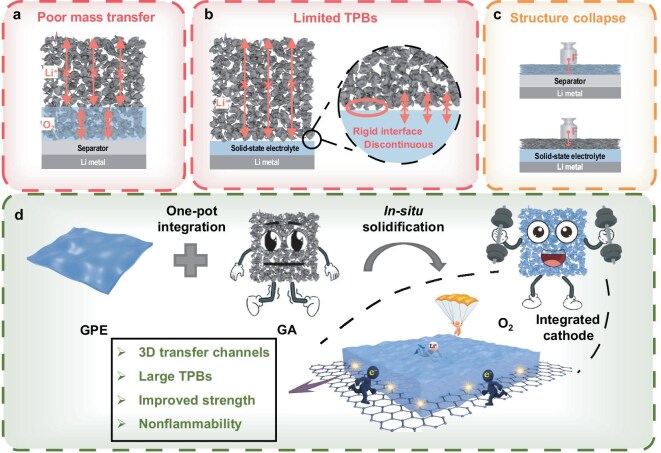
Schematic illustration of (a–c) challenges in lithium-oxygen batteries (LOBs) with thick cathodes, and (d) the advantages of the gel polymer electrolyte (GPE) integration strategy.

In LOBs, electrochemical reactions involve three-phase interactions requiring Li^+^ transport, e^−^ transport, and O_2_ supply [5,6]. Therefore, only three-phase boundaries (TPBs) are electroactive in cathodes, making them crucial performance-determining factors for LOBs [7,8]. In recent years, thick cathodes with higher active material loading have been explored to minimize the percentage of non-active portions and meet evolving application requirements [9–11]. However, constructing TPBs in these thick cathodes is quite challenging. Due to gravity, commonly used liquid electrolytes (LEs) tend to accumulate at the bottom of the thick cathode, obstructing O_2_ permeation, while being absent at the top, resulting in a lack of Li^+^ transfer channels (Fig. 1a). The fluidity of LEs prevents them from forming TPBs uniformly across the entire thick cathode, thus hindering complete utilization of the active surface. Solid-state electrolytes (SSEs), despite their rising popularity for enhanced safety, exacerbate the situation due to their poor adaptability. The rigid solid–solid interface between SSEs and cathodes leads to the formation of discontinuous TPBs only at the contact points (Fig. 1b). Moreover, if inert discharge products cover the cathode during discharge or lose contact with either the ionic or electronic transfer interface during recharge, the already sparse TPBs become completely ineffective [12,13]. As such, solid-state LOBs generally exhibit severe polarization, poor coulombic efficiency, low specific capacity, insufficient rate capability, and short lifespan [14,15]. Another major issue of thick cathodes is their severe structural collapse during cycling or even during the battery assembly process (Fig. 1c). To ensure effective interfacial contact, LOBs must be firmly compressed by current collectors. This makes it challenging for porous cathodes to maintain their structural integrity, particularly when dealing with fragile cathodes [16,17]. As a result, materials that are inherently suitable for LOB cathodes, such as graphene aerogel (GA), are rarely applied.

Herein, a novel *in-situ* integration method is employed, where a gel polymer electrolyte (GPE) serves as an SSE to encapsulate and *in-situ* solidify on the surface of a GA (Fig. 1d), forming robust TPBs with an extensive ionic and electronic dual-conducting network across the entire thick GA (∼2 mm). Within this shared porous architecture, directional transport channels are established: e^−^ travel along the continuous GA skeleton, while Li^+^ migrate through the GPE matrix that permeates the pores. Owing to the favorable adaptability of the GPE and the weak interfacial interactions between GPE and GA, this segregated yet cooperative pathway enables rapid charge transfer and a stable interface. Furthermore, the integrated GPE provides auxiliary structural support to the fragile GA, helping to preserve its porous structure that is indispensable for O_2_ transfer. With the large and uniform TPBs successfully integrated in the cathode, our quasi-solid-state LOBs exhibited commendable cycle performance and outstanding rate capability, achieving an excellent areal capacity of 34.6 mAh cm^−2^ and a remarkable gravimetric capacity of 19 000 mAh g^−1^ based on the cathode’s total weight. This work demonstrates a breakthrough in establishing continuous three-phase transport pathways throughout thick cathodes, unlocking their full potential for practical high-energy-density battery applications.

## RESULTS AND DISCUSSION

**Figure 2.**
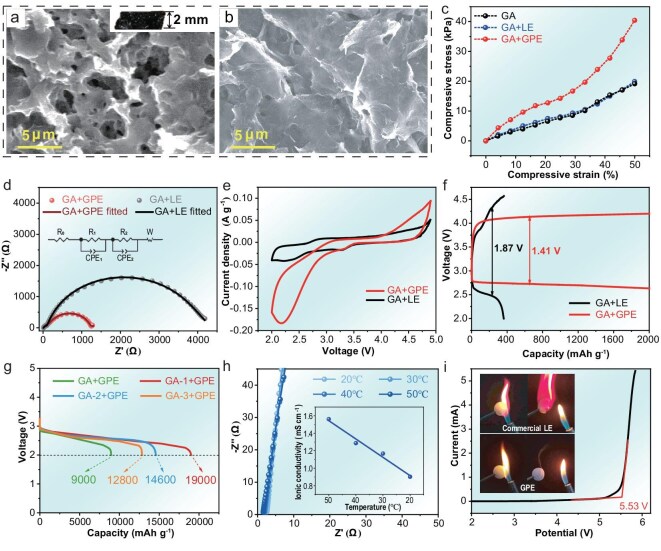
Scanning electron microscopy (SEM) images of (a) GA + GPE and (b) GA + LE after battery assembly. Inset: optical image of GA + GPE after battery assembly. (c) Compressive strain-stress profiles of GA, GA + LE, and GA + GPE. (d) Electrochemical impedance spectroscopy (EIS) of batteries using GA + LE and GA + GPE before discharge. (e) CV curves of batteries using GA + LE and GA + GPE. (f) Discharge-charge profiles of batteries using GA + LE and GA + GPE with a capacity limitation of 2000 mAh g^−1^. (g) Full discharge profiles of batteries using GA + GPE, GA-1 + GPE, GA-2 + GPE, and GA-3 + GPE. (h) Temperature-dependent ionic conductivity of the GPE. (i) LSV curve of the GPE. Inset: flame retardancy tests of the commercial LE and GPE.

An innovative *in-situ* integration strategy to address mass transfer issues in lithium-oxygen batteries (LOBs) with thick cathodes was proposed. A solid-state gel polymer electrolyte (GPE) was directly integrated into a graphene aerogel (GA) cathode structure, resulting in a composite (referred to as GA + GPE) with a thickness of ∼2 mm (Fig. 2a inset). Following the *in-situ* solidification of the GPE, a large and interconnected 3D electronic and ionic dual-conducting network was successfully constructed (Fig. 2a). The GA core provided efficient e^−^ transfer channels, while the GPE coating offered high-performance Li^+^ transfer channels. Furthermore, the GPE provided strong structural support for the GA, significantly improving its resistance to deformation (Fig. S1). As a result, even after compression during battery assembly, the porous structure of the GA remained intact. These pores greatly facilitated rapid O_2_ diffusion and accommodated discharge products. In contrast, the GA infiltrated with liquid electrolyte (referred to as GA + LE) exhibited complete structural collapse after compression, with its 3D skeleton almost entirely flattening into a 2D structure (Fig. 2b, Fig. S1). The enhancement in mechanical strength is further demonstrated in Fig. 2c, where GA + GPE exhibited significantly smaller compressive strain than GA + LE, while the strain of GA + LE remained close to that of the pristine GA.

The GA + GPE exhibited a significantly lower mass transfer resistance of 1027 Ω as shown in Fig. 2d, indicative of a high transfer efficiency of Li^+^ and O_2_ owing to the wide network of three-phase boundaries (TPBs). In contrast, the GA + LE exhibited a much higher resistance of 3780 Ω due to severe structural collapse leading to fewer available transfer channels for both Li^+^ and O_2_. The physical significance of each element in the equivalent circuit (Fig. 2d inset) is explained as follows: R_0_ represents the ohmic resistance of the battery, which includes the intrinsic electronic resistance of the electrode materials and the contact resistance at various interfaces. R_1_ and CPE_1_ are associated with the charge-transfer resistance and corresponding non-ideal capacitance, respectively, reflecting the kinetics of electrochemical reactions at both the anode and cathode interfaces. R_2_ and CPE_2_ correspond to the mass-transfer resistance and related phase element, describing the diffusion limitations of species such as Li⁺ and O_2_ within the electrode structure. The sum of R_0_, R_1_, and R_2_ represents the total resistance of the battery system, and it varies as anticipated throughout a full cycle [18].

**Figure 3.**
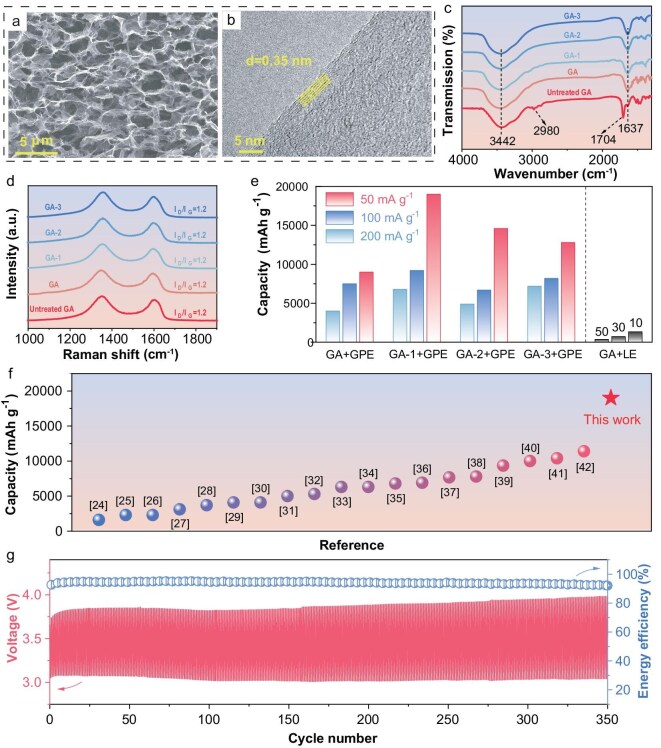
(a) SEM and (b) Transmission electron microscopy (TEM) images of GA. (c) Fourier transform infrared (FTIR) and (d) Raman spectra of GA treated at different temperatures. (e) Rate capability of batteries using GA + LE, GA + GPE, GA-1 + GPE, GA-2 + GPE, and GA-3 + GPE. (f) Comparison of discharge capacity with works listed in Table S1. (g) Cycling performance of battery using GA + GPE.

The electrochemical kinetic performances for both oxygen reduction reaction (ORR) and oxygen evolution reaction (OER) of GA + GPE and GA + LE were evaluated by cyclic voltammetry (CV) tests. As shown in Fig. 2e, the GA + GPE demonstrated markedly superior kinetics with lower onset overpotentials and higher current densities in both the anodic and cathodic scans. Such a battery with GA + GPE exhibited a lower potential gap of 1.41 V during the discharge-charge cycle with a limited capacity of 2000 mAh g^−1^, while GA + LE showed a higher value of 1.87 V and could merely discharge to 360 mAh g^−1^ (Fig. 2f). Furthermore, as shown in Fig. 2g, the GA + GPE cathode delivered an excellent full discharge capacity of 9000 mAh g^−1^ at a current density of 50 mA g^−1^. By optimazing the heat treatment temperature of the GA, its discharge performance was further enhanced. The detailed optimization process of the GA + GPE is presented in Fig. S2. Among the optimized samples, GA-1 with a heat treatment at 700°C exhibited a remarkable areal capacity of 34.6 mAh cm^−2^ and a notable gravimetric capacity of 19 000 mAh g^−1^ (calculated based on the total cathode weight), with both values approaching the highest reported in the literature. The commendable discharge performance of GA + GPE could be attributed to the integration of GPE, which significantly mitigated TPB loss, along with the excellent properties of the GPE itself. As shown in Fig. 2h, the GPE exhibited an ionic conductivity of 1.0 mS cm^−1^ at 20°C, increasing to 1.6 mS cm^−1^ at 50°C. The activation energy was determined from the slope of the linear fit of ln(σ) versus 1000/T, yielding a value of 0.147 eV. This activation energy quantitatively confirms the favorable ion transport kinetics within our GPE. Additionally, the GPE also demonstrated impressive electrochemical stability, remaining stable up to 5.56 V (Fig. 2i), which was well beyond the operating voltage range of LOBs. Moreover, it is nonflammable, as shown in the inset of Fig. 2i, significantly reducing safety concerns associated with high-energy-density batteries [19]. The microstructure of the GA treated at different temperatures is shown in Fig. 3a and Fig. S3. The GA exhibited a highly porous structure with macropores of ∼2 μm in diameter, and the pore structure remained unaffected by temperature variations. Transmission electron microscopy (TEM) images (Fig. 3b and Fig. S4) revealed that the GA consisted of ∼6 stacked graphene layers with an interlayer spacing of 0.35 nm. Brunauer-Emmett-Teller (BET) analysis via N_2_ adsorption measurements indicated that the GA possessed a high specific surface area of 354 m^2^ g^−1^ (Fig. S5).

Fourier transform infrared (FTIR) spectroscopy was employed to investigate the evolution of the GA’s surface composition under different heat treatment temperatures, elucidating the mechanism behind the capacity improvement achieved through temperature optimization. During the hydrothermal reaction, graphene oxide and ethylenediamine formed amine- and oxygen-rich organic species, ultimately generating a passivation layer on the GA surface. Subsequent heat treatment progressively decomposed this passivation layer (as evidenced by TGA weight loss in Fig. S6), exposing the underlying active sites. As shown in Fig. 3c, all samples exhibited a broad absorption peak at 3442 cm^−1^, corresponding to N–H and O–H stretching vibrations. The C–H stretching peaks near 2980 cm^−1^ diminished significantly with increasing temperature, indicating a dehydrogenation process [20]. Additionally, the oxygen-rich group vibrations at 1704 cm^−1^ gradually weakened and red-shifted, while the C=C stretching peak at 1637 cm^−1^ intensified, suggesting the decomposition of the passivation layer and partial restoration of sp^2^-hybridized domains due to the heat treatment [21]. Energy-dispersive spectroscopy (EDS) analysis further confirmed this trend (Fig. S7), revealing a temperature-dependent decrease in nitrogen and oxygen content alongside an increase in carbon concentration. In contrast, Raman spectra (Fig. 3d) showed no significant changes in the D and G bands, maintaining a consistent I_D_/I_G_ ratio of 1.2. This indicated that only the surface passivation layer decomposed while the graphene skeleton and crystallinity remained unchanged across all treatment temperatures [22]. The ORR activities of the thermally treated GA samples were evaluated using a rotating disk electrode (RDE) as shown in Fig. S8. Heat-treated samples consistently exhibited enhanced ORR performance, showing higher onset potentials and greater limiting current densities than pristine GA. This improvement in ORR activity directly correlated with decomposition of the passivation layer and the consequent enhancement in electronic conductivity of the materials [23].

The superiority of the GPE integration strategy is further demonstrated by the rate capabilities of batteries as shown in Fig. 3e and Fig. S9. The GPE-integrated GA cathodes exhibited excellent electrochemical performances, particularly at high current densities. When tested at 50 mA g^−1^, the GA samples prepared at 600, 700, 800, and 900°C integrated with GPE delivered discharge capacities of 9000, 19 000, 14 600, and 12 800 mAh g^−1^, respectively. Even when the current density was increased to 200 mA g^−1^, the discharge capacities were maintained at 4000, 6800, 4900, and 7200 mAh g^−1^, respectively. In contrast, GA + LE could only achieve a discharge capacity of 360 mAh g^−1^ at 50 mA g^−1^. Even when the current density was reduced to 10 mA g^−1^, the discharge capacity was merely 1300 mAh g^−1^. Neither the current density nor the discharge capacity of GA + LE was comparable to those of the GPE-integrated cathodes. For comparison, Fig. 3f and Table S1 showcase some typical works of graphene-based cathodes, highlighting that the performance of our GPE-integrated cathode was remarkable. The superiority of the GPE integration strategy could be summarized as two key aspects. First, the GPE provided structural support for the cathode, enabling the utilization of free-standing but fragile materials in LOBs. Free-standing cathodes, which lack extra non-active and hefty substrates, have significant advantages in specific capacity but are typically hindered by their mechanical strength in practical operation. Second, the integrated GPE constructed three-phase transfer channels throughout a thick cathode, making it feasible to use thick cathodes with higher areal capacity in LOBs. Furthermore, the GPE-integrated cathode also exhibited a pretty good cycle performance as shown in Fig. 3g. The battery demonstrated 350 cycles without significant voltage decay while maintaining a high energy efficiency of ∼93%.

**Figure 4.**
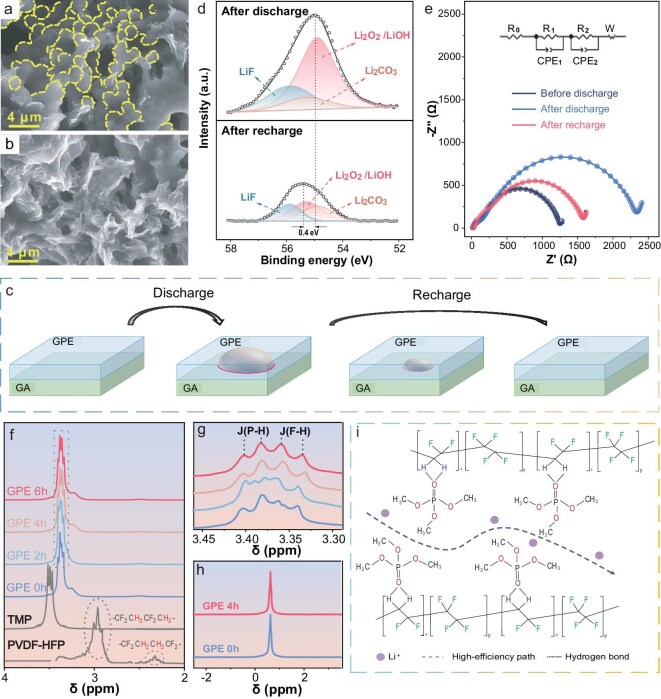
SEM images of GA + GPE (a) after discharge and (b) after recharge. (c) Schematic illustration of the Li_2_O_2_ evolution process in GA + GPE during a discharge-recharge cycle. (d) XPS spectra of GA + GPE after discharge and recharge. (e) EIS of battery using GA + GPE at different states. *In-situ* NMR spectra of the GPE solidification process: (f) ^1^H, (g) magnification of rectangle region in (f), (h) ^7^Li. (i) Schematic illustration of Li^+^ transfer mechanism in GPE.

The evolution of discharge products in the GPE-integrated cathode (the lithium side) was followed using SEM. Upon full discharge, numerous spheroidal particles with diameters ranging from 400 to 1000 nm were uniformly sandwiched between the GPE and GA (Fig. 4a). Together with Fig. S11a (the oxygen side), the consistent presence of discharge products on both sides of the electrode confirms that the GPE maintains continuous contact with the graphene layers throughout the cycling process. Notably, these discharge products vanished upon full recharge without apparent residue, and the cathode structure returned to its original highly porous morphology (Fig. 4b). The integrated cathode maintained its structural integrity throughout the discharge-recharge cycle, demonstrating exceptional mechanical strength that effectively prevented electrode collapse. This robust integrated architecture ensured uninterrupted mass transport pathways, thereby mitigating premature battery failure. Furthermore, the GPE exhibited excellent interfacial adaptability, as evidenced by its ability to tightly reconform to the GA surface during cycling. As schematically illustrated in Fig. 4c, the GPE dynamically adjusted its morphology in response to Li_2_O_2_ formation and decomposition, maintaining continuous contact with the TPB (highlighted in magenta). This adaptive behavior effectively suppressed interfacial failure commonly encountered in quasi-solid-state LOBs, contributing to the observed electrochemical reversibility.

The chemistry of the discharge products was analyzed using X-ray photoelectron spectroscopy (XPS) as shown in Fig. 4d. The primary discharge product was identified as Li_2_O_2_, with Li_2_CO_3_ and LiOH likely formed as side products [43,44]. The combined fraction of Li_2_O_2_ and LiOH is ∼68.54%, with Li_2_CO_3_ alone accounting for 11.19%, and LiF constituting 20.36%. LiF formed from the reaction between the FEC additive and O_2_^−^ species was found to be a crucial component in stabilizing the solid electrolyte interphase (SEI) [45,46]. After a full recharge, the peak corresponding to Li_2_O_2_/LiOH significantly weakened, indicating the decomposition of Li_2_O_2_ and confirming excellent reversibility of the GPE-integrated cathode.

Electrochemical impedance spectroscopy (EIS) of the battery using the GPE-integrated cathode was measured at various states: before discharge, after a full discharge, and after a full recharge, as shown in Fig. 4e. The total resistance of the battery before discharge was 1178 Ω, which rapidly increased to 2344 Ω after a full discharge because of the deposition of inert discharge products. After a full recharge, the total resistance decreased to 1407 Ω, also demonstrating the excellent reversibility of the battery. The slight increase in resistance after recharge is commonly observed in LOBs, induced by the formation of an SEI layer [47,48].

**Figure 5.**
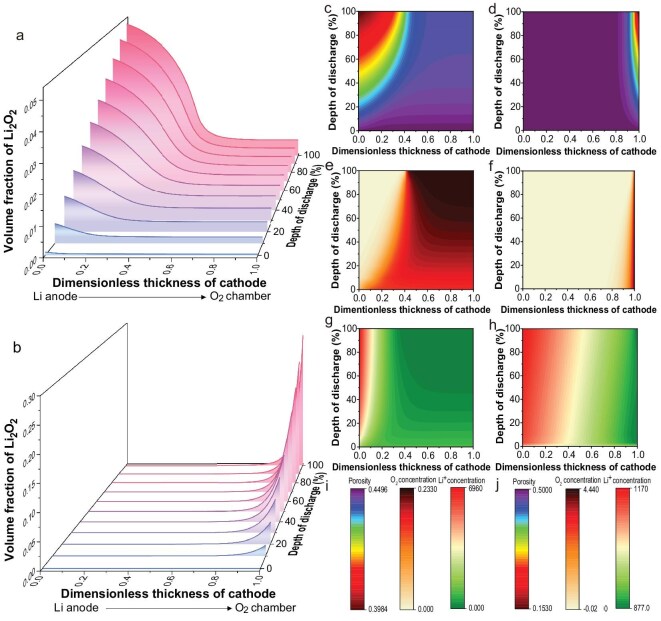
Numerical simulation of the evolution of (a, b) Li_2_O_2_ distribution, (c, d) porosity, (e, f) O_2_ concentration, and (g, h) Li^+^ concentration, along with (i, j) their corresponding color bars, as a function of discharge depth in batteries using (a, c, e, g, i) GA + GPE and (b, d, f, h, j) GA + LE.

Previous studies indicate that the solidification time of GPE decreases as the lithium salt content increases [49]. *In-situ* nuclear magnetic resonance (NMR) spectroscopy was utilized to monitor the solidification process of the GPE and to elucidate the Li^+^ transfer mechanism [50]. As shown in Fig. 4f, trimethyl phosphate (TMP) and poly(vinylidene fluoride-co-hexafluoropropylene) (PVDF-HFP) exhibited characteristic multiplet peaks due to J-coupling of P–H and F–H [51,52]; no chemical shift variation was detected for the C–H bonds, confirming that the integration between GPE and GA is mediated by weak interactions. These multiple peaks gradually overlapped upon mixing within the GPE and eventually formed a quartet peak, indicating significant interactions between TMP and PVDF-HFP (Fig. 4g). Simultaneously, the ^1^H peak of TMP downshifted from 3.51 to 3.40 ppm, while the ^1^H peak of PVDF-HFP upshifted from 2.85 to 3.33 ppm, suggesting an increase in electron density around TMP and a decrease around PVDF-HFP. In contrast, the ^7^Li peak showed no notable variation before and after the solidification of the GPE (Fig. 4h), suggesting that the chemical environment surrounding Li^+^ remained unchanged. Based on these NMR results, the Li^+^ transfer mechanism was deduced and schematically illustrated in Fig. 4i. Within the GPE, the highly electronegative O atoms in the TMP molecules could withdraw electrons from the -CH_2_ groups of the PVDF-HFP, enabling the formation of hydrogen bonds. This interaction traps TMP molecules within the polymer matrix, forming a highly efficient path for Li⁺ transfer and thereby enhancing ionic conductivity. A numerical simulation model was introduced to elucidate the mass transfer processes in batteries using GA + GPE and GA + LE, to shed light on the success of the GPE integration strategy. The model construction and validation details are presented in the Method section and illustrated in Fig. S12 and Fig. S13 of the supporting information. As shown in Fig. 5a, the successful construction of three-phase transfer channels in the GA + GPE resulted in a distribution of Li_2_O_2_ throughout the cathode, denser near the Li anode and thinner near the O_2_ chamber. In contrast, the Li_2_O_2_ distribution in the GA + LE was much narrower, with Li_2_O_2_ aggregating only near the O_2_ chamber (Fig. 5b). The evolution of cathode porosity was consistent with the variation of Li_2_O_2_ (Fig. 5c and Fig. 5d). This comparison clearly indicated that the entire region of the GA + GPE was available for electrochemical reactions, while the GA + LE only had a very limited reactive region.

The evolution of O_2_ and Li^+^ concentrations provided further insights. In the GA + GPE, the O_2_ concentration remained relatively steady, whereas the Li^+^ concentration varied dramatically (Fig. 5e and Fig. 5g). As discharge progressed, the Li^+^ concentration gradient increased rapidly, rising near the Li anode side (L side) and declining near the O_2_ chamber side (O side). This suggested that Li^+^ tended to accumulate at the L side due to transfer resistance, resulting in higher Li^+^ consumption at the O side compared to its replenishment, and the discharge process would eventually halt when Li^+^ was depleted. Conversely, in the GA + LE, while Li^+^ transfer was ensured, O_2_ transfer was severely impeded, with O_2_ concentration decreasing by >92% in the majority of the region after just 0.5% depth of discharge (Fig. 5f and Fig. 5h).

## CONCLUSION

The practical challenges inherent in thick cathodes for lithium-oxygen batteries (LOBs) have been successfully overcome through an innovative gel polymer electrolyte (GPE) integration strategy. The integrated GPE not only provided crucial mechanical support to the fragile cathodic structure thus maintaining optimal oxygen diffusion channels within the porous cathode, but also allowed construction of an extensive dual-conducting network for both e^−^ and Li^+^ throughout the entire thick cathode (∼2 mm), thereby fully unlocking the high energy density potential of thick cathodes. The resulting integrated cathode featured well-organized and continuous three-phase boundaries (TPBs), delivering remarkable electrochemical performance with an areal capacity of 34.6 mAh cm^−2^ and a gravimetric capacity of 19 000 mAh g^−1^ (based on the total cathode weight). Additionally, the GPE exhibited excellent adaptive capability to dynamically accommodate Li_2_O_2_ formation and decomposition, which effectively maintained TPB availability and alleviated interfacial degradation—a common limitation in quasi-solid-state LOBs—leading to an impressive cycle life of 350 cycles. Numerical simulation further validated the effectiveness and superiority of this integrated approach, demonstrating its strong potential for practical implementation. This work not only establishes a general methodology for implementing thick cathodes in battery systems but also provides a conceptual framework for integrating delicate materials into advanced energy storage devices.

## Supplementary Material

nwag134_Supplemental_File
