# Supplementary material for: Sharing electronic and ionic transfer channels for high-energy-density and stable quasi-solid-state lithium-oxygen battery
Source: Natl Sci Rev. 2026 Mar 5;13(9):nwag134. doi: 10.1093/nsr/nwag134 (PMC13220746; doi:10.1093/nsr/nwag134)
Supplement: nwag134_Supplemental_File [file nwag134_supplemental_file.pdf]

Supporting Information

# Sharing Electronic and Ionic Transfer Channels for High-Energy-Density and Stable Quasi-Solid-State Lithium-Oxygen Battery

Yuanguo Wu[1,2,6], Zhuojun Zhang[3], Jiaqi Wang[2], Hongtao Qu[1], Jing Li[1], Liuxi Yang[1], Amanda Kale[1], Xikun Zhang[1], Xiangyu Wen[2], Zhihong Wang[2], Zhe Lü[2], Yu Li[4,*], Peng Tan[3,*], Xingbao Zhu[5,*] and Bao-Lian Su[1,4,*]

1 Laboratory of Inorganic Materials Chemistry (CMI)
University of Namur
61 rue de Bruxelles, Namur, B-5000, Belgium
E-mail: bao-lian.su@unamur.be

2 School of Physics
Harbin Institute of Technology
2 Yikuang Road, Harbin, 150000, China

3 Department of Thermal Science and Energy Engineering
University of Science and Technology of China
96 Jinzhai Road, Hefei 230000, China
E-mail: pengtan@ustc.edu.cn

4 State Key Laboratory of Advanced Technology for Materials Synthesis and Processing
Wuhan University of Technology
122 Luoshi Road, Wuhan, 430070, China
E-mail: yu.li@whut.edu.cn and bao-lian.su@unamur.be

5 School of Physics
Beijing Institute of Technology
9 Liangxiang East Road, Beijing, 102488, China
E-mail: zhuxingbao008@163.com

6 Gotion High-tech Co., Ltd.
599 Daihe Road, Hefei 230026, China
E-mail: wuyuanguo@gotion.com.cn

## METHODS

### SYNTHESIS OF GRAPHENE AEROGEL (GA) AND GEL POLYMER ELECTROLYTE (GPE)

The GA was fabricated as follows: few-layer graphene oxide (GO) powder (Suzhou Hengqiu Tech. Inc.) was dispersed in deionized water by sonication at a concentration of 1 mg $mL^{-1}$. A 2 mL GO solution and 100 μL ethanediamine (Aladdin) were sealed in a Teflon-lined stainless-steel autoclave and subjected to hydrothermal treatment at 180 °C for 12 h. After undergoing a freeze-drying process, a cylindrical untreated GA was obtained. This untreated GA was further heated at various temperatures (600, 700, 800 and 900 °C) for 2 h under an Ar atmosphere (99.999%), and the resulting samples were denoted as GA, GA-1, GA-2, and GA-3, respectively.

The GPE was prepared as follows: 100 mg PVDF-HFP (Mw≈400000, Sigma-Aldrich) was dissolved in 1 mL trimethyl phosphate (TMP, Aladdin) and 100 μL fluoroethylene carbonate (FEC, Aladdin) by magnetic stirring. Then, 1 mmol lithium bis-trifluoromethanesulfonimide (LiTFSI, Aladdin) was added to the above solution.

### CHARACTERIZATION

The scanning electron microscope (SEM) images and energy dispersive spectroscopy (EDS) were taken with a Hitachi S-4700 field emission scanning electron microscope. The transmission electron microscope (TEM) was imaged by a JEM· 2100F microscope. Raman spectra were recorded by a Nanobase XperRam Compact Raman system using an excitation wavelength of 532 nm. X-ray photoelectron spectroscopy (XPS) was performed on a Thermo Fisher eacalab250xi X-ray photoelectron spectrometer. To remove residual lithium salts and electrolyte, the electrodes were rinsed with anhydrous dimethyl ether (DME) three times, with careful attention paid to distinguishing the electrolyte-facing side from the air-facing side. Fourier transform infrared spectroscopy (FTIR) was carried out on a Thermo Nicolet iS10 spectrometer. Thermo-gravimetric analysis (TGA) was measured on a TA SDT2960 thermal analyser under an Ar atmosphere with a heating speed of 5 °C $min^{-1}$. The mechanical strength was recorded by an Instron 3344 test machine. The specific surface area was analysed by liquid nitrogen sorption measurements based on the Brunauer-Emmett-Teller method (Micromeritics ASAP2460).

### BATTERY ASSEMBLY AND ELECTROCHEMICAL TESTS

Batteries were assembled in an Ar-filled glove box (Braun, Labstar 1250X780) with $O_2$ and $H_2O$ levels below 0.5 ppm. The configuration is a GA sample, a fresh Li metal, a cellulose separator (NKK, MPF30AC-100), and a certain amount of the GPE. The mass of the cathode GA is approximately 0.9 mg. For this mass, 100 μl of GPE are used for integration. For comparison, batteries were also assembled with a commercial liquid electrolyte (1 M LiTFSI/TEGDME) in the same way. After assembly, the batteries were purged with pure $O_2$ for 3-5 minutes and then sealed for testing under a constant $O_2$ pressure of 1 atm at 25 °C. The galvanostatic charge and discharge experiments were performed on a battery testing system (Neware, CT-2800W) with a voltage limit of 2.0-5.0 V. At the same time, long-term cyclic experiments were also carried out, with a fixed specific capacity limit of 500 mAh $g^{-1}$ (based on the mass of GA) at a current density of 50 mA $g^{-1}$. Electrochemical performances were tested by an electrochemical workstation (Chenhua, CHI660E). Electrochemical impedance spectroscopy (EIS) was measured in a frequency range of 0.1-$10^5$ Hz with an AC voltage amplitude of 5 mV. Ionic conductivity was measured using an SS symmetric cell and recorded in a temperature range of 20-50 °C. Linear sweep voltammetry (LSV) measurements were carried out with a scan rate of 0.1 mV $s^{-1}$. Rotating disk electrode (RDE) measurements were carried out on the ALS RRDE-3A system in an $O_2$-saturated 0.1 M KOH electrode with a scan rate of 10 mV $s^{-1}$. A saturated calomel electrode and a Pt wire were used as the reference electrode and counter electrode, respectively. In-situ $^1H$ In-situ nuclear magnetic resonance (NMR) measurements were acquired on a 500 MHz JEOL ECZR NMR spectrometer equipped with a 5 mm inverse broadband probe. A coaxial NMR tube setup was employed to eliminate the influence of deuterated solvent on the sample. The inner tube was filled with d6-DMSO, which served both as a field lock and an external reference. The electrolyte precursors were mixed and then injected into the outer tube. NMR spectra were acquired at two-hour intervals throughout the

experiment. Ex-situ static $^7Li$ NMR spectra of the gel polymer electrolyte were collected on a 600MHz JEOL NMR spectrometer using 3.2 mm MAS probe.

## NUMERICAL SIMULATION MODEL DEVELOPMENT

### ELECTROCHEMICAL KINETICS

The local current of GA ($j_{lc}$) and Li ($j_{la}$) electrodes are expressed by the Butler-Volmer kinetic equation.

GA electrode: $$\frac{j_{lc}}{nF} = -i_{0,c}\frac{c_{O_2}}{c_{O_2,ini}}\left(\frac{c_{Li^+}}{c_{Li^+,ini}}\right)^2 exp\left(-\eta_c\frac{\alpha nF}{RT}\right) \quad (1)$$

Li electrode: $$j_{la} = i_{0,a}\left\{exp\left[\eta_a\frac{(1-\alpha)nF}{RT}\right] - exp(\eta_a\frac{-\alpha nF}{RT})\right\} \quad (2)$$

where $c_{O_2}$ and $c_{Li^+}$ are oxygen and Li ion concentration along the GA surface, and the subscript in denoted initial value. The overpotential of the GA electrode ($\eta_c$) is defined as:

$$\eta_c = \phi_s - \Delta\phi_{s,film} - \phi_l - E_c^{eq} \quad (3)$$

$$\Delta\phi_{s,film} = j_{lc}R_{Li_2O_2}\varepsilon_{Li_2O_2} \quad (4)$$

in which $\phi_s$ and $\phi_l$ are solid and liquid potential, respectively, $E_c^{eq}$ is the theoretical equilibrium potential of the oxygen reduction reaction, $\Delta\phi_{s,film}$ is the voltage drop resulting from $Li_2O_2$ forming on the GA surface, $R_{Li_2O_2}$ is electrical resistivity across the $Li_2O_2$ film, and $\varepsilon_{Li_2O_2}$ is the volume fraction of $Li_2O_2$.

## MASS TRANSPORT

### GEL POLYMER ELECTROLYTE (GPE)

In the GPE impregnated GA cathode, gaseous oxygen successively transfer within two phases as schematically shown in Fig. S10. First, oxygen transfers along with gas channels, then it passes through the oxygen/GPE interface and diffuses toward the GA surface. The transfer of gaseous oxygen can be described by Fick's law:

$$\mathbf{N}_{O_2,g} = -D_{O_2,g}\nabla c_{O_2,g} \quad (5)$$

where $D_{O_2,g}$ is the diffusion coefficient of oxygen in the gas channels, $c_{O_2,g}$ is gaseous oxygen concentration. The dynamic process for oxygen passing through the gas/GPE interface can be simplified by Henry's law, which is determined by the saturated concentration of oxygen in the GPE:

$$c_{O_2,e} = H \cdot c_{O_2,g} \quad (6)$$

where H is solubility factor, $c_{O_2,g}$ is the oxygen concentration in the GPE phase at the gas/GPE interface.

Mass conservation of species i (oxygen or Li ion) in the GPE is expressed as:

$$\frac{\partial(c_i)}{\partial t} = -\nabla \cdot \mathbf{N}_i + S_i \quad (7)$$

where $\mathbf{N}_i$ is the molar flux of oxygen and $S_i$ is the reaction source term. For oxygen, the above can be written as:

$$\mathbf{N}_{O_2,e} = \frac{aD_{O_2,e}(c_{O_2}-c_{O_2,e})}{\delta_e} \quad (8)$$

$$S_{O_2} = -\frac{aj_{lc}}{2F} \quad (9)$$

where a is the specific surface area, $\delta_e$ is the thickness of the GPE, and $D_{O_2,e}$ is the diffusion coefficient of oxygen in the GPE.

With the increase of the GPE thickness, its ability for oxygen transfer decreases while that for Li ion transfer enhances. The transport rate for Li ion can be described by the effective diffusion coefficient ($D_{Li^+}^{eff}$), which is greatly contributed by the volume fraction of the GPE ($\varepsilon_e$):

$$D_{Li^+,e}^{eff} = \varepsilon_e^{1.5}D_{Li^+,e} \quad (10)$$

$$\varepsilon_e = \gamma \delta_e \tag{11}$$

where $\gamma$ is the correlation coefficient to describe the relationship between the GPE thickness and the volume fraction in the cathode. Therefore, an optimal GPE thickness exists to tune the trade-off between oxygen and Li ion transport. The molar flux and reaction source term are written as:

$$\mathbf{N}_{Li^+,e} = -D_{Li^+,e}^{eff} \nabla c_{Li^+} + \frac{\mathbf{i}_l t_+}{F} \tag{12a}$$

$$S_{Li^+} = -\frac{a j_{lc}}{F} \tag{12b}$$

$Li_2O_2$ is formed at GA/GPE interface and the volume fraction is expressed as:

$$\varepsilon_{Li_2O_2} = \frac{M_{Li_2O_2}}{\rho_{Li_2O_2}} \int_0^{+\infty} \frac{a j_{lc}}{2F} dt \tag{13}$$

where $M_{Li_2O_2}$ and $\rho_{Li_2O_2}$ represent the molar weight and the density of $Li_2O_2$, respectively. During discharging, the formed $Li_2O_2$ squeezes the gas channels thus causing a decrease of porosity, but the volume fraction of GPE is considered to remain constant. The relationship between volume fractions are described as follows:

$$\varepsilon_g = \varepsilon_{g,ini} - \varepsilon_{Li_2O_2} \tag{14}$$

$$\varepsilon_g + \varepsilon_e + \varepsilon_{GA} + \varepsilon_{Li_2O_2} = 1 \tag{15}$$

where $\varepsilon_g$ is the volume fraction of the gas channel, i.e., porosity. Dynamic change of specific surface area (a) for the GA/GPE interface is related to the pore structure and $Li_2O_2$, which can be expressed by:

$$a = a_{ini} \left[ 1 - \left( \frac{\varepsilon_{Li_2O_2}}{\varepsilon_{g,ini} + \varepsilon_{e,ini}} \right)^{0.5} \right] \tag{16}$$

**LIQUIS ELECTROLYTE (LE)**

In the LE (LiTFSI/TEGDME) impregnated GA cathode, gaseous oxygen is dissolved at the LE/oxygen interface, where boundary condition is set to oxygen saturation concentration ($cO_{2,s,l}$). Equation (7) is used to describe the mass conservation of species i in the LE. The molar flux of Li ion in the LE is calculated as the same way as in the GPE, while diffusion of oxygen in the LE is calculated by Fick's law:

$$\mathbf{N}_{O_2,l} = -D_{O_2,l}^{eff} \nabla c_{O_2} \tag{17}$$

$$D_{O_2,l}^{eff} = \varepsilon_l^{1.5} D_{O_2,l} \tag{18}$$

where $D_{O_2,l}$ is the diffusion coefficient of oxygen in the LE. $\varepsilon_l$ is the volume fraction of the LE, i.e., porosity, which is rewritten as:

$$\varepsilon_l = \varepsilon_{l,ini} - \varepsilon_{Li_2O_2} \tag{19}$$

$$\varepsilon_l + \varepsilon_{GA} + \varepsilon_{Li_2O_2} = 1 \tag{20}$$

The specific surface area is rewritten as:

$$a = a_{ini} \left[ 1 - \left( \frac{\varepsilon_{Li_2O_2}}{\varepsilon_l} \right)^{0.5} \right] \tag{21}$$

**CHARGE CONSERVATION**

Charge conservation between the solid and solution phases is described by:

$$\nabla \mathbf{i}_l = -\nabla \mathbf{i}_s = aj \tag{22}$$

The ionic electric potential and electrolyte potential are related to the solid and solution currents, respectively, which can be written as:

$$\mathbf{i}_l = -\kappa_{eff} \nabla \phi_l - \frac{2RT\kappa_{eff}}{F}(1 - t_+)\left(1 + \frac{\partial \ln f}{\partial \ln c_{Li^+}}\right) \ln c_{Li^+} \tag{23}$$

$$\mathbf{i}_s = -\sigma_{eff}\nabla\phi_s \tag{24}$$

where $\kappa_{eff}$ is the effective ionic conductivity, $\kappa_{eff} = \varepsilon_{GA}^{1.5}\kappa$. $\sigma_{eff}$ is the effective electric conductivity, $\sigma_{eff} = \varepsilon_e^{1.5}\sigma$. $f$ is activity coefficient of the Li salt. The used parameters are listed in Table S2.

**MODEL VALIDATION**

As shown in Fig. S11, the simulated discharge profile matches well with the experimental data, which validates that the present model is reasonable and capable for further numerical analyses.

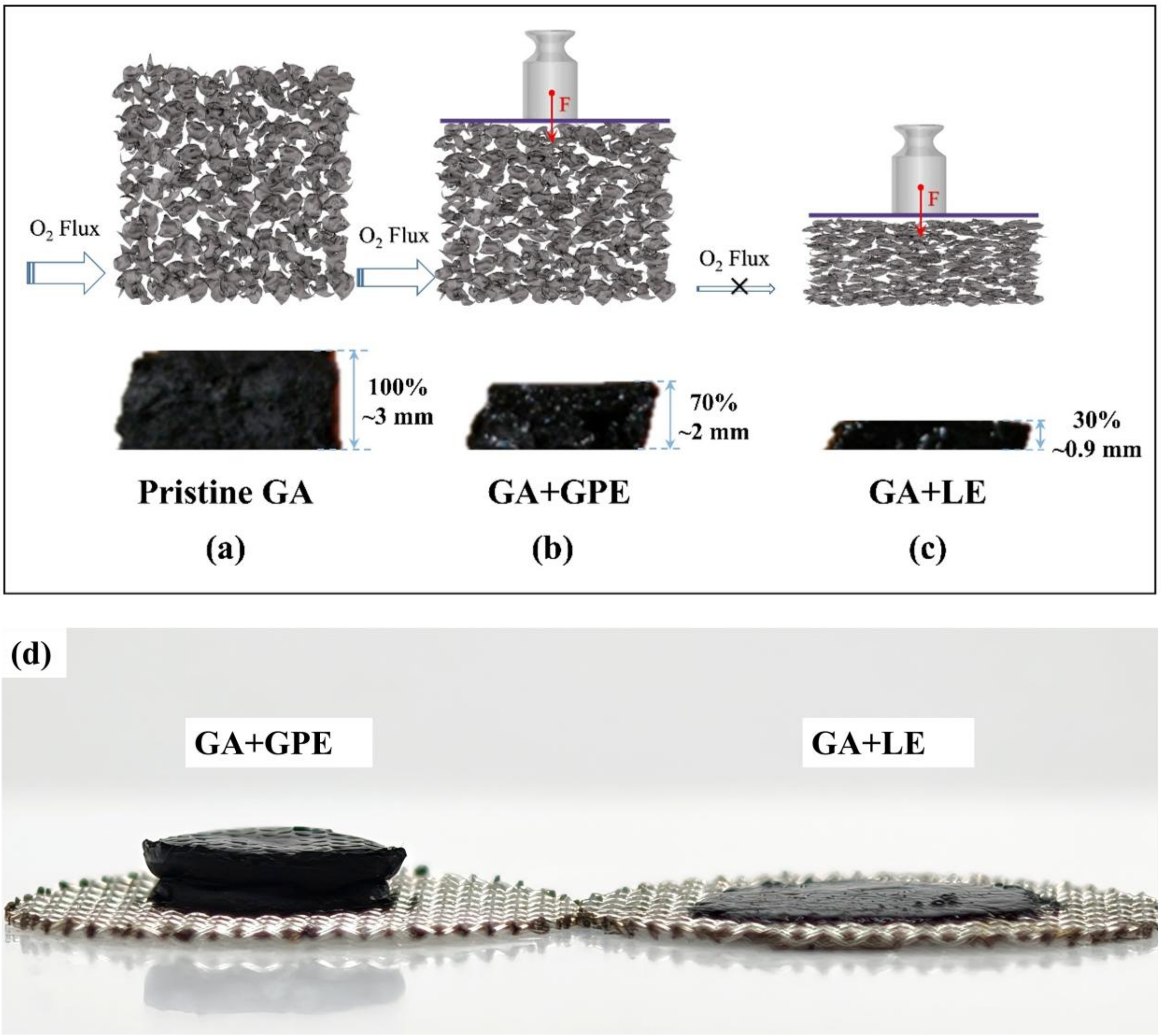


**Figure S1.** Schematic and optical images of (a) pristine GA, (b) GA+GPE after battery assembly, and (c) GA+LE after battery assembly. (d) The optical images of GA+GPE and GA+LE after long-term cycling.

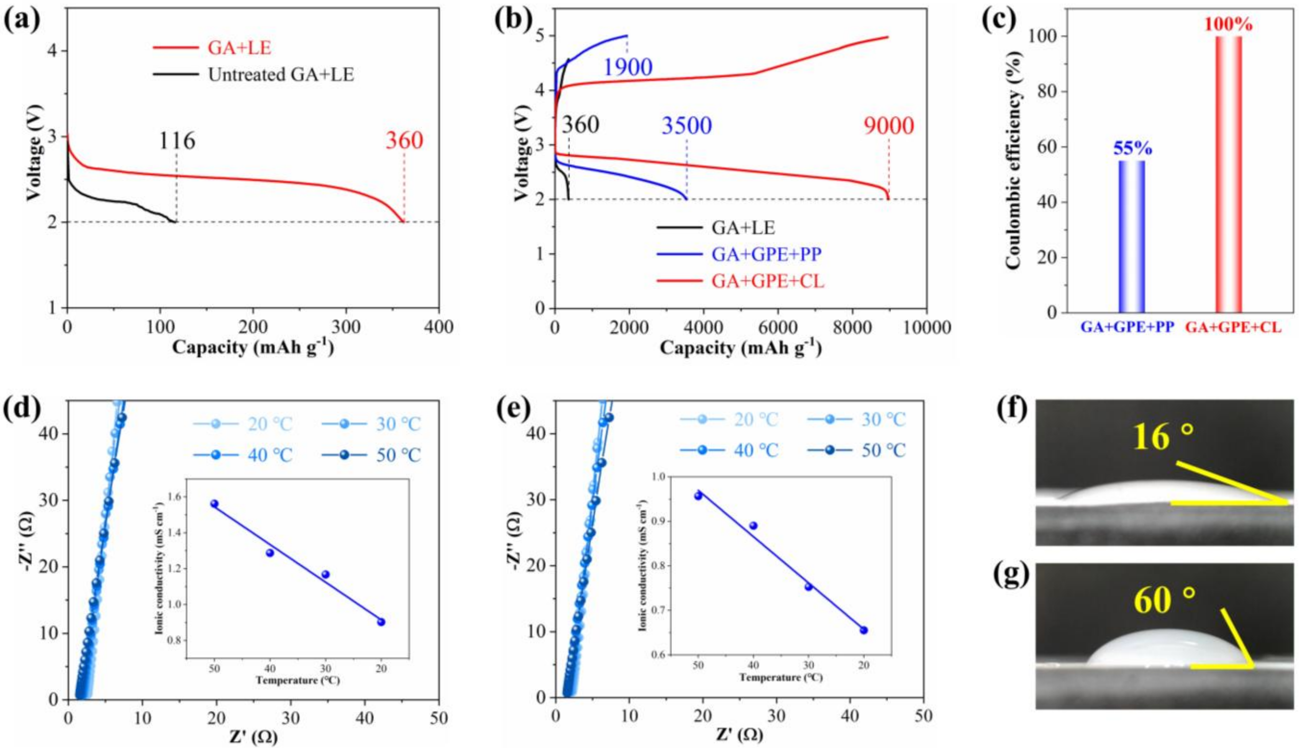


**Figure S2.** Discharge curves of batteries using GA+LE and untreated GA+LE. (b) Discharge-recharge curves of batteries using GA+LE, GA+GPE+PP, and GA+GPE+CL with (c) corresponding coulombic efficiency. (d, e) Temperature-dependent ionic conductivity of GPE+CL and GPE+PP. Contant angle images of a GPE drop on (f) CL and (g) PP separators.

As shown in Fig. S2a, the battery using GA exhibited an enhanced performance compared to the one using untreated GA, suggesting that heat treatment improved capacity of the cathodes. As shown in Fig. S2b, the battery using GA+GPE and a polypropylene (PP) separator delivered a significantly improved discharge capacity of 3500 mAh $g^{-1}$, which was an order of magnitude higher than that of GA+LE. However, the introduction of GPE led to a critical issue during recharge: the voltage increased sharply and reached the cutoff voltage of 5.0 V. This could be ascribed to the incompatibility between the pore size of separator and the viscosity of GPE. As shown in Fig. S2g, the contact angle between a GPE drop and a PP separator was 60 °, indicating poor wettability. In contrast, the GPE exhibited good wettability to a cellulose (CL) separator with a significantly smaller contact angle of 16° (Fig. S2f). The improved compatibility resulted in higher ionic conductivity as shown in Fig. S2d-S2e. The ionic conductivity of the GPE with different separators was measured by clamping a GPE-impregnated separator between two stainless steel plates, on the basis of the equation $\sigma = S/(RL)$, where L was the thickness of the GPE-impregnated separator, R was the resistance obtained from the Nyquist pilot, and S was the geometric surface area of the separator. The temperature-dependent ionic conductivities were obtained by AC impedance spectroscopy conducted from 20 to 50 °C. The ionic conductivity of GPE+PP separator was approximately $7.1 \times 10^{-4}$ S $cm^{-1}$ at room temperature. As the temperature increases, the value gradually rose and reached $9.6 \times 10^{-4}$ S $cm^{-1}$ at 50 °C. When using a CL separator, the ionic conductivity exhibited an increase of 44% at room temperature with a value of $1.0 \times 10^{-3}$ S $cm^{-1}$ and reached $1.6 \times 10^{-3}$ S $cm^{-1}$ at 50 °C. Noticeably, the GPE could not be used directly without a separator, so we calculated a more practical conductivity herein based on a separator integrated with GPE rather than GPE itself. After optimizing the separator, the charge potential of the battery decreased obviously and the coulombic efficiency improved from 55% to 100% (Fig. S2c). Meanwhile, the discharge capacity increased substantially to 9000 mAh $g^{-1}$ as shown in Fig. S2b red line.

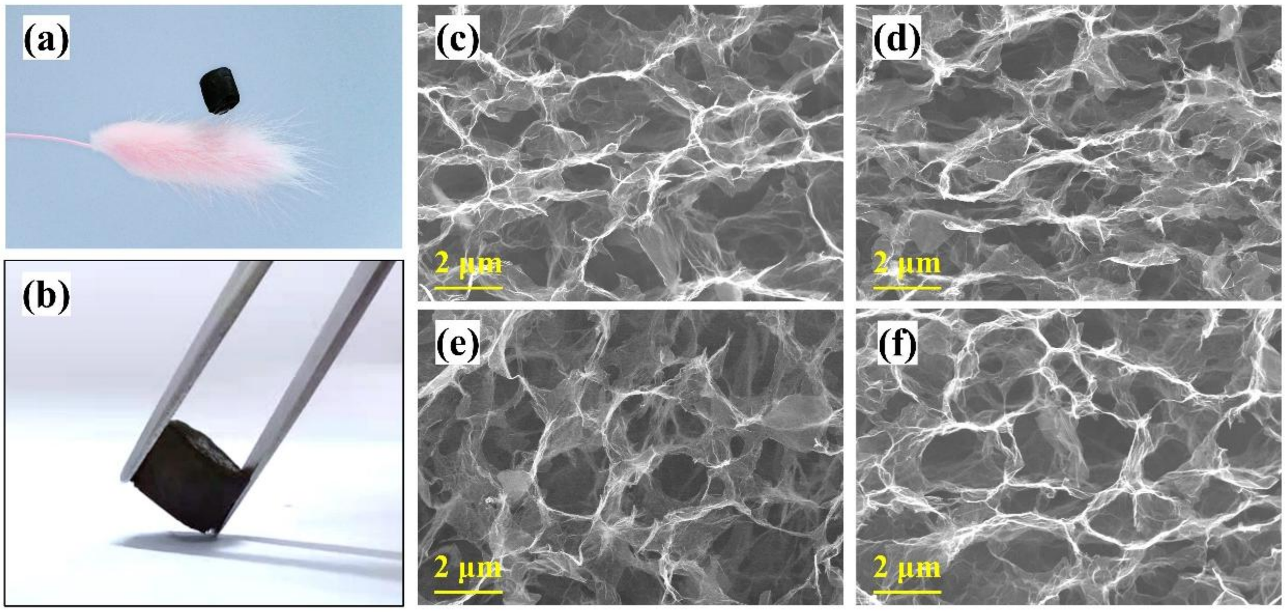


**Figure S3.** (a, b) Optical images of GA. SEM images of (c) GA, (d) GA-1, (e) GA-2, and (f) GA-3.

As shown in Fig. S3a-S3b, the GA exhibited a cylindrical shape, was light enough to rest on a setaria viridis, and was mechanically robust enough to be handled with tweezers.

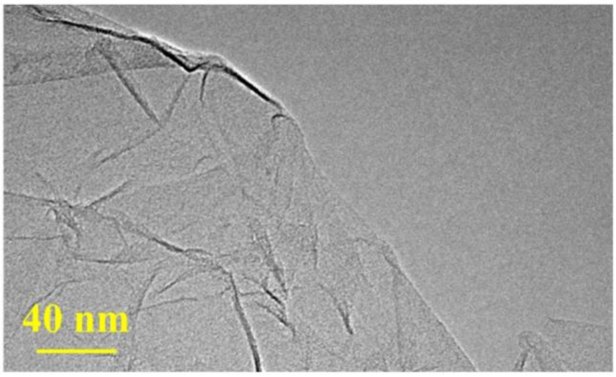


**Figure S4.** TEM image of GA.

Wrinkles and folded regions along graphene surface could be clearly observed, which not only enlarged the surface area but also improved the elastic stiffness of the GA.

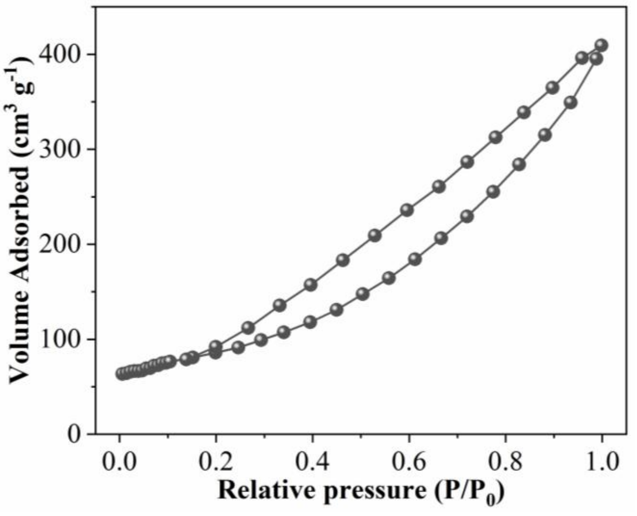


**Figure S5.** BET nitrogen adsorption-desorption isotherms of GA.

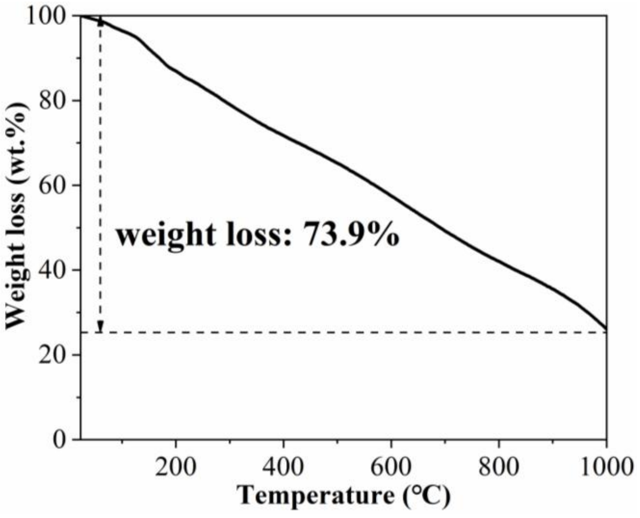


**Figure S6.** TGA curve of GA.

After heat treatment, the passivated layer on the GA surface decomposed, demonstrating as the 73.9% weight loss in the TGA curve, which enabled surface active sites to be fully exposed and would consequently stimulate electrochemical reaction.

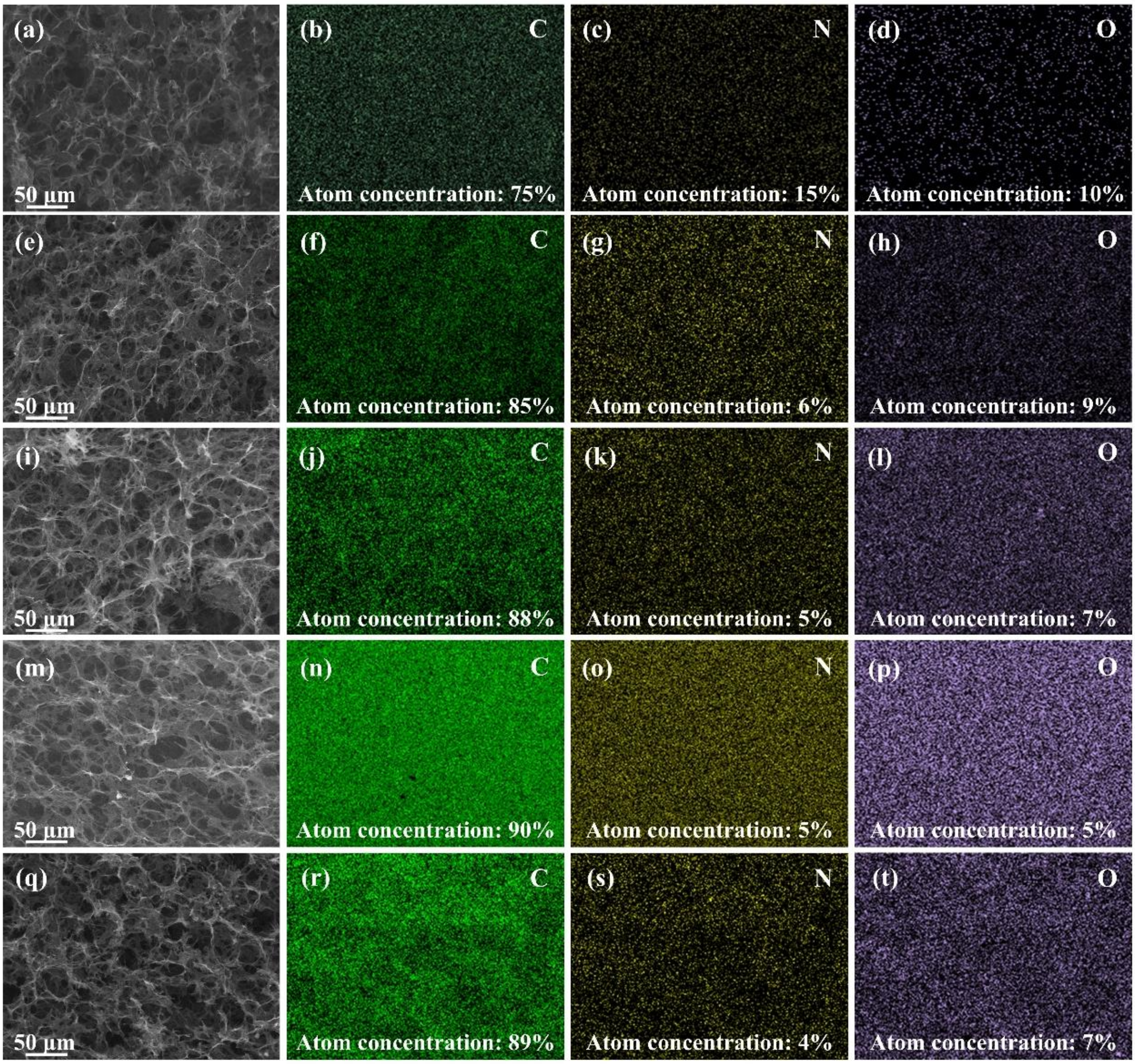


**Figure S7.** SEM-EDS mapping of (a-d) untreated GA, (e-h) GA, (i-l) GA-1, (m-p) GA-2, and (q-t) GA-3.

The atom concentration of carbon increased from 75% to 90%, while that of nitrogen and oxygen decreased, which further demonstrated the decomposition of the organic layer and thus suggested the activation of GA.

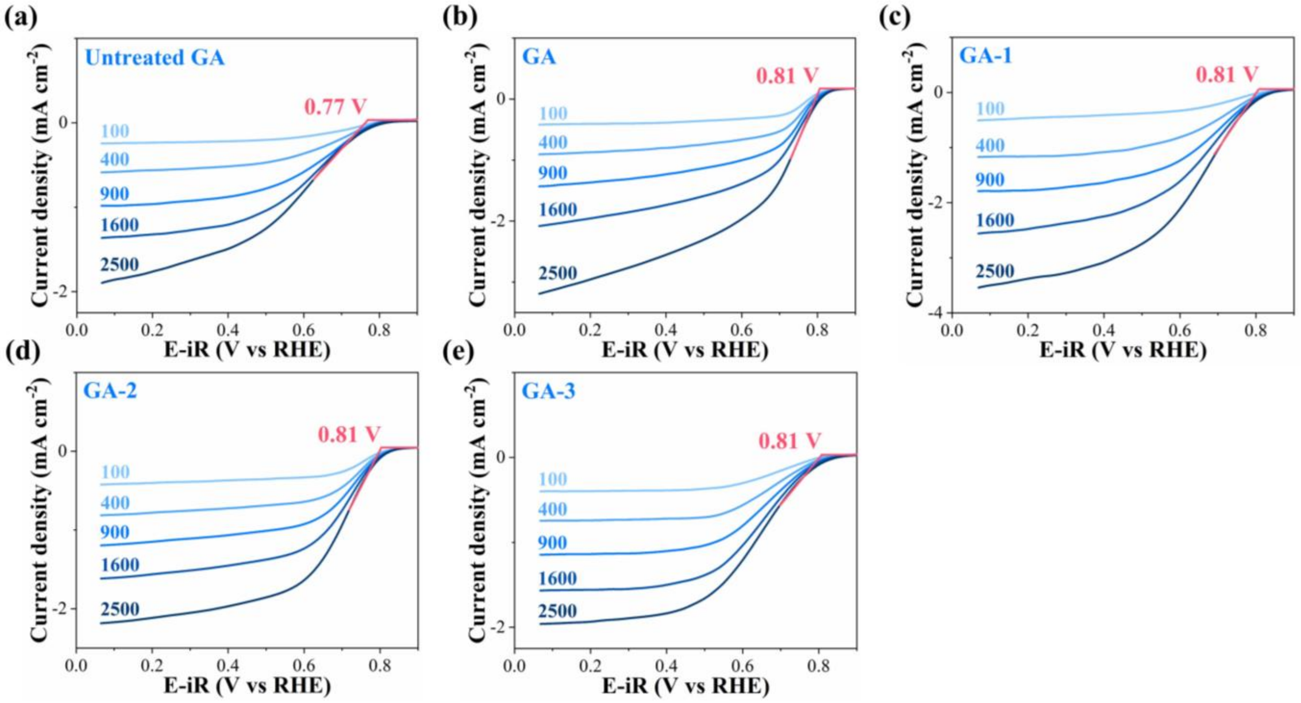


**Figure S8.** RDE curves of (a) untreated GA, (b) GA, (c) GA-1, (d) GA-2, and (e) GA-3 with various rotation speeds.

The intrinsic ORR activity of the as-treated samples was characterized by recording LSV curves on a rotating disk electrode (RDE). As shown in Fig. S8a, the untreated GA delivered an onset potential of approximately 0.77 V (vs. RHE), compared with 0.81 V of heat-treated samples (Fig. S8b-S8e). Moreover, the limited current densities of the heat-treated samples were higher than that of the untreated GA, and the GA-1 delivered the greatest value of 3.54 mA cm$^{-2}$ at 2500 rpm. Briefly, heat-treated samples owing to fewer electrochemically active groups (e.g., amorphous carbon and C=O bonds) exhibited superior ORR activity, which was contrary to the accredited opinion that those unsaturated bonds were favorable for the ORR. The reason could be ascribed as follows: Unlike the GA skeleton built through a self-assembly process where graphene sheets were connected by C=C bonds and π-conjugated structures, the contact between amorphous carbons and the GA surface was fragile, which probably magnified their defect of sluggish electronic conductivity and even covered merits for the ORR.

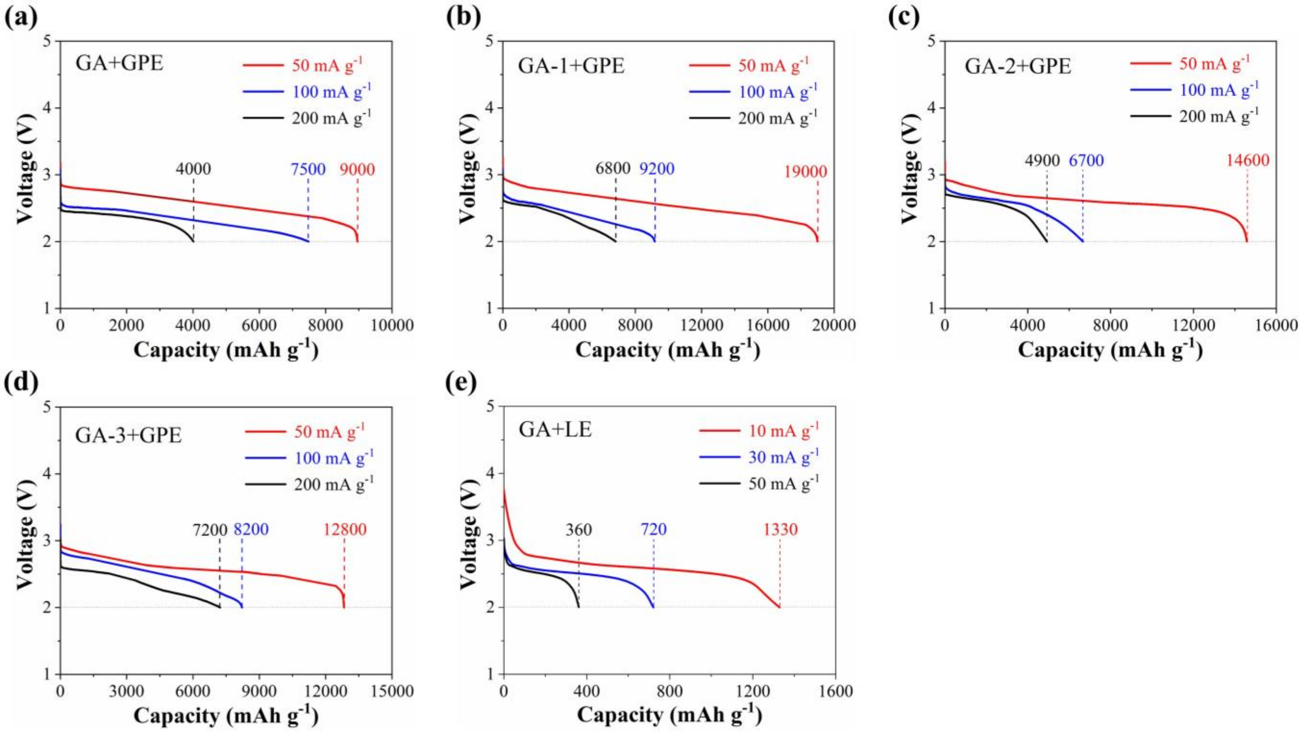


**Figure S9.** Rate capability of batteries using (a) GA+GPE, (b) GA-1+GPE, (c) GA-2+GPE, (d) GA-3+GPE, and (e) GA+LE.

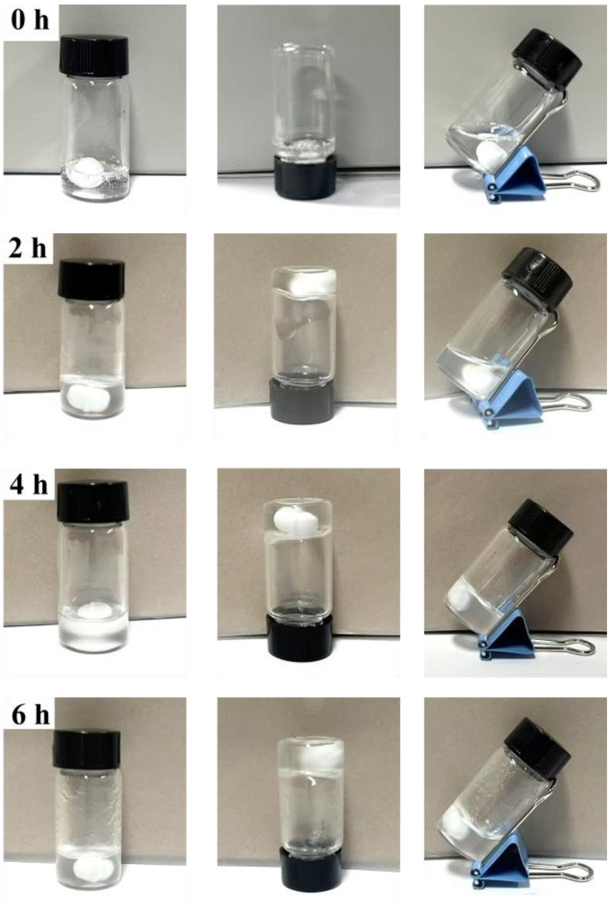


**Figure S10.** Digital photos of the GPE solidification process at different stages.

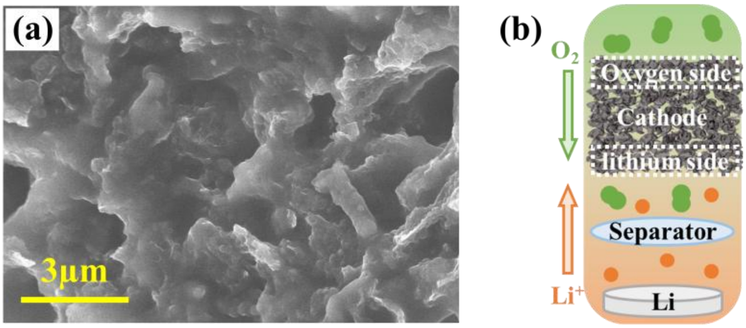


**Figure S11.** (a) SEM images of the oxygen side of GA+GPE after discharge, and (b) schematic illustration of lithium-oxygen battery.

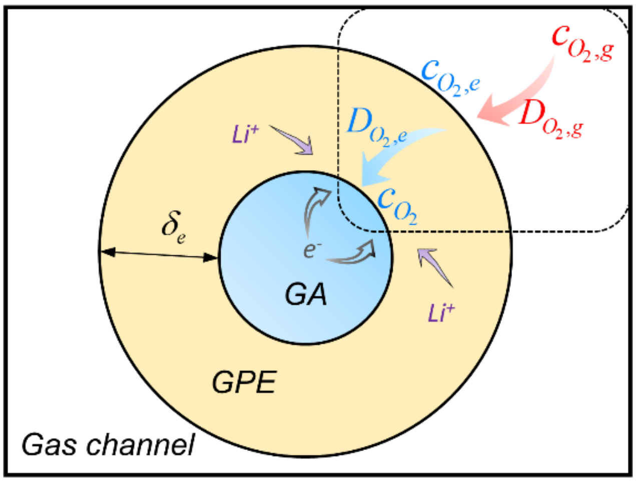


**Figure S12.** Schematic illustration of the mass transport processes in the GA+GPE cathode.

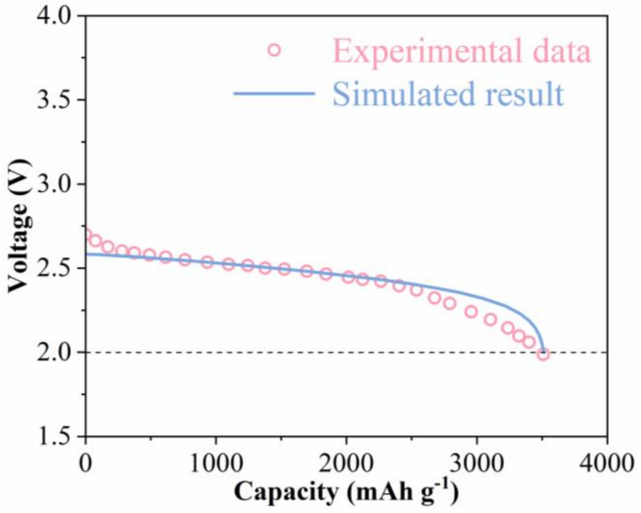


**Figure S13.** The comparison between the simulated discharge profile and the experimental data with a cut-off voltage of 2.0 V.

**Table S1.** Full discharge performances of some previously reported LOBs with graphene-based cathodes in comparison to this work.

| Materials | Electrode preparation | Cathodic substrate | Specific current density | | Specific capacity | | Reference |
|---|---|---|---|---|---|---|---|
| | | | Gravimetric ($mA\ g^{-1}$) | Areal ($mA\ cm^{-2}$) | Gravimetric ($mAh\ g^{-1}$) | Areal ($mAh\ cm^{-2}$) | |
| Graphene nanosheets | Slurry coating | Aluminum mesh | -- | 0.1 | 5300 | -- | [1] |
| Graphene nanoribbons | Slurry coating | PP separator | 210 | -- | 6300 | -- | [2] |
| Graphene nanosheets | Slurry coating | Stainless steel mesh | 50 | 0.1 | 2300 | -- | [3] |
| Reduced graphene oxides | Slurry coating | Ni mesh | -- | 0.1 | 5000 | -- | [4] |
| N-doped graphene | Slurry coating | Ni foam | 50 | | 13 500 | -- | [5] |
| Holey graphene | Slurry coating | Carbon paper | 300 | -- | 7800 | -- | [6] |
| Graphene nanoplatelets & graphene oxides | Vacuum filtration | None | 200 | -- | 6910 | -- | [7] |
| Holey Graphene | Dry compression | None | 20 | 0.1 | 7670 | 37.3 | [8] |
| Carbonized melamine foam-graphene | Template-directed method | None | 50 | -- | 4100 | -- | [9] |
| Porous graphene foam | Template-directed method | None | 57 | 0.02 | 9350 | 2.8 | [10] |
| Hierarchically porous graphene | Template-directed method | Ni foam | -- | -- | 6300 | --- | [11] |
| 3D porous graphene | Hydrothermal self-assembly | Carbon paper | 200 | -- | 10300 | -- | [12] |
| N-doped graphene aerogel | Hydrothermal self-assembly | Ni foam | 100 | -- | 1600 | -- | [13] |
| Graphene aerogel | Hydrothermal self-assembly | Ni foam | -- | 0.05 | 4100 | -- | [14] |
| N-doped 3D graphene | Hydrothermal self-assembly | Carbon paper | 500 | 0.2 | 10000 | 4 | [15] |
| N&S co-doped graphene nanosheets | Hydrothermal self-assembly | Carbon paper | 100 | 0.1 | 11400 | 11.4 | [16] |
| 3D graphene | Hydrothermal self-assembly | None | 50 | -- | 2300 | -- | [17] |
| Graphene hydrogel | Hydrothermal self-assembly | None | 100 | -- | 3120 | -- | [18] |
| Graphene sponge | Hydrothermal self-assembly | None | -- | 0.1 | 6800 | -- | [19] |
| **GA-1** | **Hydrothermal self-assembly** | **None** | **50** | **0.05** | **19000** | **34.6** | **This work** |

**Table S2.** Parameters used in simulation.

| Parameters | Symbol | Value | Unit | Reference |
|---|---|---|---|---|
| Anodic exchange current density | $i_{0,a}$ | 1 | A $m^{-2}$ | [20] |
| Cathodic exchange current density | $i_{0,c}$ | $3\times10^{-6}$ | A $m^{-2}$ | Fitted |
| Symmetry factor | $\alpha$ | 0.5 | 1 | [20] |
| Thickness of GA electrode | $L_{pos}$ | 2 | mm | Measured |
| Thickness of GPE | $\delta_{\varepsilon}$ | 150 | nm | Measured |
| Density of $Li_2O_2$ | $\rho_{Li_2O_2}$ | 2140 | kg $m^{-3}$ | Measured |
| Molecular weight of $Li_2O_2$ | $M_{Li_2O_2}$ | $45.88\times10^{-3}$ | kg $mol^{-1}$ | Measured |
| Electrical resistivity across $Li_2O_2$ film | $R_{Li_2O_2}$ | 50 | $\Omega\cdot m^2$ | [20] |
| Initial volume fraction of gas channel | $\varepsilon_{g,ini}$ | 0.45 | 1 | Calculated |
| Initial volume fraction of GA electrode | $\varepsilon_{GA,ini}$ | 0.1 | 1 | Calculated |
| Initial volume fraction of GPE | $\varepsilon_{e,ini}$ | 0.45 | 1 | Calculated |
| Initial volume fraction of TEGDME | $\varepsilon_{l,ini}$ | 0.9 | 1 | Calculated |
| Correlation coefficient | $\gamma$ | 0.003 | $nm^{-1}$ | Fitted |
| Initial specific surface area | $a_{ini}$ | $2.1\times10^{6}$ | $m^{-1}$ | Calculated |
| $O_2$ diffusion coefficient in gas channel | $D_{O_2,g}$ | $1.81\times10^{-5}$ | $m^2$ $s^{-1}$ | [21] |
| $O_2$ diffusion coefficient in GPE | $D_{O_2,e}$ | $1.2\times10^{-15}$ | $m^2$ $s^{-1}$ | Fitted |
| $O_2$ diffusion coefficient in TEGDME | $D_{O_2,l}$ | $2.17\times10^{-10}$ | $m^2$ $s^{-1}$ | [22] |
| $O_2$ solubility factor | $H$ | 0.0058 | 1 | Fitted |
| Initial $O_2$ concentration in gas channel | $cO_{2,g,ini}$ | 40.34 | mol $m^{-3}$ | Calculated |
| Saturated $O_2$ concentration in TEGDME | $cO_{2,s,l}$ | 4.43 | mol $m^{-3}$ | [22] |
| $Li^+$ diffusion coefficient in GPE | $D_{Li^+,e}$ | $7.4\times10^{-13}$ | $m^2$ $s^{-1}$ | Fitted |
| $Li^+$ diffusion coefficient in TEGDME | $D_{Li^+,l}$ | $8\times10^{-11}$ | $m^2$ $s^{-1}$ | [22] |
| Electric conductivity of GA | $\sigma$ | 100 | S $m^{-1}$ | [23] |
| Ionic conductivity of GPE | $\kappa$ | $10^{-3}$ | S $m^{-1}$ | Measured |
| Derivative of natural logarithm of Li salt activity to $Li^+$ | $\frac{\partial \ln f}{\partial \ln c_{Li^+}}$ | -1.03 | 1 | [24] |
| Transference number of $Li^+$ | $t_+$ | 0.43 | 1 | [24] |

**Table S3.** Fit parameter table of EIS of batteries using GA+LE and GA+GPE before discharge.

| Materials | CPE-T | | R0 | | R1 | | R2 | |
|---|---|---|---|---|---|---|---|---|
| | Value | Error% | Value | Error% | Value | Error% | Unit | Error% |
| GA+GPE | $7.640 \times 10^{-6}$ | 3.43% | 11.69 | 1.92% | 139.2 | 1.89% | 1027 | 0.41% |
| GA+LE | $6.029 \times 10^{-6}$ | 6.41% | 12.92 | 1.66% | 71.33 | 2.68% | 3780 | 1.21% |

## REFERENCES


1. Feng N, Mu X, Zheng M *et al.* A multi-layered $Fe_2O_3$/graphene composite with mesopores as a catalyst for rechargeable aprotic lithium–oxygen batteries. *Nanotechnology* 2016; **27**: 365402.
2. Yin J, Carlin J, Kim J *et al.* Synergy between metal oxide nanofibers and graphene nanoribbons for rechargeable lithium-oxygen battery cathodes. *Adv Energy Mater* 2014; **5**: 1401412.
3. He M, Zhang P, Liu L *et al.* Hierarchical porous nitrogen doped three-dimensional graphene as a free-standing cathode for rechargeable lithium-oxygen batteries. *Electrochim Acta* 2016; **191**: 90–97.
4. Arul A, Pak H, Moon K *et al.* Metallomacrocyclic–carbon complex: A study of bifunctional electrocatalytic activity for oxygen reduction and oxygen evolution reactions and their lithium-oxygen battery applications. *Appl Catal B Environ* 2018; **220**: 488–496.
5. Palani R, Wu Y, Wu S, et al. Cobalt nanoclusters Deposit on Nitrogen-Doped graphene Sheets as bifunctional electrocatalysts for high performance lithium–Oxygen batteries. J Colloid Interface Sci 2025, **680**: 845-858.
6. Zhang J, Chen X, Lei Y *et al.* Highly rechargeable lithium oxygen batteries cathode based on boron and nitrogen co-doped holey graphene. *Chem Eng J* 2022; **428**: 131025.
7. Kim D, Kim M, Kim D *et al.* Flexible binder-free graphene paper cathodes for high-performance Li-$O_2$ batteries. *Carbon* 2015; **93**: 625–635.
8. Lin Y, Moitoso B, Martinez-Martinez C *et al.* Ultrahigh-capacity lithium–oxygen batteries enabled by dry-pressed holey graphene air cathodes. *Nano Lett* 2017; **17**: 3252–3260.
9. Zhang P, Wang R, He M *et al.* 3D hierarchical Co/CoO-graphene-carbonized melamine foam as a superior cathode toward long-life lithium oxygen batteries. *Adv Funct Mater* 2016; **26**: 1354–1364.
10. Ji X, Zhu X, Huang X *et al.* In situ fabrication of porous graphene electrodes for high-performance lithium-oxygen batteries. *Int J Hydrog Energy* 2018; **43**: 16128–16135.
11. Yu W, Shen Z, Yoshii T e*t al.* Hierarchically porous and minimally stacked graphene cathodes for high-performance lithium–oxygen batteries. *Adv Energy Mater* 2024; **14**: 2303055.
12. Liu Y, Meng W, Gao Y *et al.* Binder-free three-dimensional porous graphene cathodes via self-assembly for high-capacity lithium–oxygen batteries. *Nanomaterials* 2024; **14(9)**: 754.
13. Hsieh T, Tsou Y, Chen J. Iron phthalocyanine supported on 3D nitrogen-doped graphene aerogel as an electrocatalyst for non-aqueous Li−$O_2$ batteries. *Electrochim Acta* 2019; **295**: 490–497.
14. Li L, Chen C, Su J *et al.* Three-dimensional $MoS_x$ ($1<x<2$) nanosheets decorated graphene aerogel for lithium–oxygen batteries. *J Mater Chem A* 2016; **4**: 10986–10991.
15. Liu M, Sun K, Zhang Q *et al.* Rationally designed three-dimensional N-doped graphene architecture mounted with Ru nanoclusters as a high-performance air cathode for lithium–oxygen batteries. *ACS Sustainable Chem Eng* 2020; **8**: 6109–6117.

16. Kim J, Kannan A, Woo H *et al.* A bi-functional metal-free catalyst composed of dual-doped graphene and mesoporous carbon for rechargeable lithium–oxygen batteries. *J Mater Chem A* 2015; **3**: 18456–18465.

17. Sun B, Wang B, Su D *et al.* Graphene nanosheets as cathode catalysts for lithium-air batteries with an enhanced electrochemical performance. *Carbon* 2012; **50**: 727–733.

18. Yang Y, Yin W, Wu S *et al.* Perovskite-type LaSrMnO electrocatalyst with uniform porous structure for an efficient Li–$O_2$ battery cathode. *ACS Nano* 2016; **10**: 1240–1248.

19. Yu L, Shen Y, Huang Y. Fe-N-C catalyst modified graphene sponge as a cathode material for lithium-oxygen battery. *J Alloys Compd* 2014; **595**: 185–191.

20. Sahapatsombut U, Cheng H, Scott K. Modelling the micro–macro homogeneous cycling behaviour of a lithium–air battery. *J Power Sources* 2013; **227**: 243–253.

21. Gwak G and Ju H. Three-dimensional transient modeling of a non-aqueous electrolyte lithium-air battery. *Electrochim Acta* 2016; **201**: 395–409.

22. Laoire C, Mukerjee S, Abraham K *et al.* Influence of nonaqueous solvents on the electrochemistry of oxygen in the rechargeable lithium−air battery. *J Phys Chem C* 2010; **114**: 9178–9186.

23. Worsley M, Pauzauskie P, Olson T *et al.* Synthesis of graphene aerogel with high electrical conductivity. *J Am Chem Soc* 2010; **132**: 14067–14069.

24. Jung C, Zhao T, An L. Modeling of lithium–oxygen batteries with the discharge product treated as a discontinuous deposit layer. *J Power Sources* 2015; **273**: 440–447.
